# Mode of conception and pregnancy outcomes at term among low‐risk nulliparous patients

**DOI:** 10.1002/pmf2.70472

**Published:** 2026-10-24

**Authors:** Gabriella D. Cozzi‐Glaser, Christina T. Blanchard, Lauren A. Pace, Lynda Ugwu, Sukhkamal Campbell, Alan T. Tita, Rachel G. Sinkey

**Affiliations:** ^1^ Department of Obstetrics and Gynecology University of Alabama at Birmingham Birmingham Alabama USA; ^2^ Center for Women's Reproductive Health University of Alabama at Birmingham Birmingham Alabama USA; ^3^ Department of Biostatistics School of Public Health University of Alabama at Birmingham Birmingham Alabama USA

**Keywords:** cesarean delivery, hypertensive disorder of pregnancy, in vitro fertilization, ovulation induction

## Abstract

**Introduction:**

Available data suggest that assisted reproductive technology (ART) is associated with adverse perinatal outcomes; however, prior studies have been confounded by multifetal gestations, preterm birth, and patient medical comorbidities. There are limited data on pregnancy outcomes among ART‐conceived low‐risk singleton pregnancies delivering at term. Thus, we aimed to measure the association between mode of conception and mode of delivery among low‐risk nulliparous patients delivering singleton gestations at term.

**Methods:**

Secondary analysis of the ARRIVE trial (NCT 01990612), a multicenter randomized, controlled, unmasked, parallel‐group trial of nulliparous patients delivering at term, March 2014–August 2017. Pregnancies were categorized by mode of conception as either ART or spontaneous. The primary outcome was cesarean delivery. Secondary outcomes included a composite of perinatal death and severe neonatal complications, and select maternal and neonatal adverse outcomes. Bivariate analyses compared baseline characteristics and outcomes by mode of conception. Primary and secondary outcomes were compared using multivariable logistic regression to generate odds ratios (ORs) and 95% confidence intervals (CIs). Sensitivity analyses by type of ART (in vitro fertilization [IVF] vs. ovulation induction [OI] with or without artificial insemination [AI]) and by embryo age were planned.

**Results:**

Of the 6106 participants in the original trial, 6084 met eligibility criteria: 157 (2.6%) conceived with ART and 5927 (97.4%) did not. Among those with ART, 103 (65.6%) underwent IVF and 54 (34.4%) conceived with OI or AI. Patients undergoing ART were noted to have an older age, differing race/ethnicity, lower body mass index (BMI), more likely to be married, employed full‐time, privately insured, and less likely to smoke cigarettes. Cesarean occurred more frequently in those with ART than those spontaneously conceived (29.9% vs. 20.2%; OR, 1.69; 95% CI, 1.20–2.40). This remained significant after adjusting for confounders (aOR, 1.68; 95% CI, 1.15–2.45) and was significant in subgroup analysis comparing IVF conception to spontaneous (31.1% vs. 20.2%; aOR, 1.78; 95% CI, 1.12–2.82). Additionally, there was no difference in hypertensive disorders of pregnancy (aOR, 1.65; 95% CI, 1.00–2.73), other maternal morbidities, and adverse perinatal composite (aOR, 0.90; 95% CI, 0.49–1.67).

**Conclusions:**

Nulliparas delivering a singleton fetus at term conceived by ART had an increased rate of cesarean delivery, with no difference in hypertensive disorders or pregnancy or neonatal outcomes.

## 1 INTRODUCTION

Pregnancies conceived by assisted reproductive technology (ART) have numerous perinatal risks, including cesarean delivery [1, 2], hypertensive disorders of pregnancy (HDP) [3], and preterm birth (PTB) [1]. However, it remains unclear whether adverse outcomes are related to the technology itself, underlying ART population differences, multifetal gestations, PTB, or other factors, as studies have demonstrated increased risks of cesarean and perinatal morbidity among patients with subfertility not undergoing ART [4] and among singletons conceived with ART [1]. Previous analyses of ART outcomes have generally included heterogeneous populations with potential confounders of multifetal gestations and PTB. Therefore, the objective of this study is to examine mode of delivery and outcomes of singleton pregnancies conceived by ART, as compared to spontaneous conception (SC), among a well‐characterized cohort of low‐risk nulliparas delivering at term.

## 2 MATERIALS AND METHODS

We conducted a secondary analysis of the multicenter, randomized, controlled, parallel group, unmasked Labor Induction versus Expectant Management in Low‐Risk Nulliparous Women (ARRIVE) trial (NCT01990612) [5]. In the parent trial, 6106 enrolled patients were randomized in 1:1 fashion to induction of labor at 39 0/7 to 39 4/7 weeks gestational age (GA) versus expectant management. Patients included in the parent trial had a viable singleton in vertex position, no known contraindication to vaginal delivery, and no planned cesarean. Included patients were considered low risk, meaning they had no high‐risk conditions, for example, fetal growth restriction, known anomaly, HDP, etc., that would have resulted in an indicated induction prior to 40 5/7 weeks. The primary perinatal composite outcome of the parent trial was not significantly different between those undergoing scheduled induction between 39 0/7 and 39 4/7 weeks of gestation and those undergoing expectant management; however, patients undergoing elective induction had a significantly lower rate of cesarean and HDP.

All participants from the original trial were eligible for this secondary analysis. Participants were excluded if the conception method was unavailable or if the neonate was diagnosed postnatally with a major anomaly. Patients were characterized by self‐reported mode of conception: ART (including in vitro fertilization [IVF], ovulation induction [OI], and/or artificial insemination [AI]) and spontaneous conception (SC).

The primary outcome was cesarean delivery rate. Secondary outcomes were broadly categorized into neonatal outcomes including a composite of neonatal morbidity (Table 2) and maternal outcomes such as HDP, chorioamnionitis, and postpartum hemorrhage (Table 3).

Maternal demographics, primary, and secondary outcomes were compared between ART and SC using Student's *t*‐tests, Chi‐square, and Fisher's exact tests as appropriate. Multivariable logistic regression was used to estimate adjusted odds ratios (aORs) and 95% confidence intervals (CIs) to assess the association between mode of conception and the primary outcome. Adjusted models included baseline characteristics differing by mode of conception (*p* < 0.05) in bivariable analysis and without multicollinearity, assessed by Pearson correlation coefficients. A similar approach estimated the association between mode of conception and dichotomous secondary outcomes. We conducted exploratory analyses to examine the association between cesarean and type of ART, comparing IVF and OI and/or AI to SC, separately. Mode of conception was compared for primary and secondary outcomes using one‐way ANOVAs, Chi‐square, and exact tests as appropriate. All analyses were performed using SAS Version 9.4 and evaluated with *p* < 0.05 as the level of significance.

## 3 RESULTS

Of 6106 participants in the original trial, 6084 were included for analysis: 157 (2.6%) conceived with ART and 5927 (97.4%) with SC (Figure 1). Among the ART cohort, 103 (65.6%) underwent IVF and 54 (34.4%) underwent OI and/or AI. ART patients were noted to have an older maternal age and a lower body mass index (BMI) at randomization (Table 1). Additionally, those undergoing ART were more likely to be White non‐Hispanic race/ethnicity, married or partnered, employed full‐time, privately insured, and less likely to smoke cigarettes (Table 1).

**FIGURE 1:**
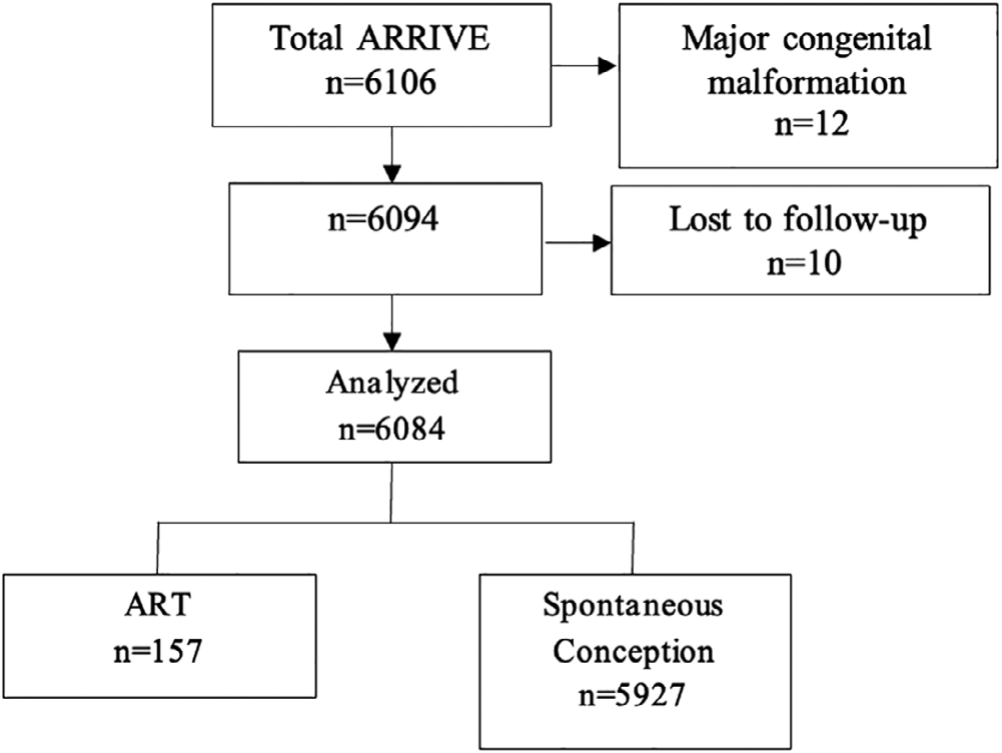
Flow diagram depicting patients identified for inclusion in final analysis.

**TABLE 1:** Baseline maternal characteristics of term among low‐risk nulliparous women delivering in the ARRIVE trial 2014–2017.

Characteristic	ART (*n* = 157)	Spontaneous conception (*n* = 5927)	*p* value
Maternal age^a^ (years)	31.2 ± 4.2	24.6 ± 4.8	<0.001
Maternal age (years)			<0.001
≤17	0 (0.0%)	161 (2.7%)	
18–25	14 (8.9%)	3589 (60.6%)	
26–29	37 (23.6%)	1116 (18.8%)	
30–34	66 (42.0%)	851 (14.4%)	
35–40	37 (23.6%)	201 (3.4%)	
≥41	3 (1.9%)	9 (0.2%)	
Age ≥ 35 years	40 (25.5%)	210 (3.5%)	<0.001
Number of previous pregnancies			0.023
0	103 (65.6%)	4505 (76.0%)	
1	40 (25.5%)	1048 (17.7%)	
2	8 (5.1%)	265 (4.5%)	
3	4 (2.6%)	72 (1.2%)	
4	1 (0.6%)	20 (0.3%)	
≥5	1 (0.6%)	17 (0.3%)	
Race/Ethnicity			<0.001
Black, non‐Hispanic	6 (3.9%)	1394 (23.7%)	
White, non‐Hispanic	120 (77.9%)	2560 (43.5%)	
Hispanic	13 (8.4%)	1655 (28.1%)	
Asian	15 (9.7%)	176 (3.0%)	
Other	0 (0.0%)	96 (1.6%)	
BMI at randomization	30.2 ± 5.5	31.6 ± 6.2	0.006
Married or living with partner	149 (94.9%)	3453 (58.3%)	<0.001
Employment status			<0.001
Employed full time	118 (76.1%)	2310 (39.1%)	
Employed part time	9 (5.8%)	682 (11.5%)	
Not employed	28 (18.1%)	2920 (49.4%)	
Private insurance	145 (92.4%)	2587 (43.7%)	<0.001
Smoked cigarettes	3 (1.9%)	461 (7.8%)	0.006
Drank alcohol	6 (3.8%)	231 (3.9%)	0.96
Treatment group			0.24
Induction of labor	86 (54.8%)	2968 (50.1%)	
Expectant management	71 (45.2%)	2959 (49.9%)	

*Note*: Data are presented as *n* (%), mean ± SD unless otherwise stated.

Abbreviation: ART, assisted reproductive technology.

^a^ Deidentified ages were omitted.

The primary outcome of cesarean occurred in 29.9% of the ART group and 20.2% of the SC group, corresponding to increased odds of cesarean (OR, 1.69; 95% CI, 1.20–2.40; Table 2). In multivariable analysis, the odds of cesarean remained higher among the ART group (aOR, 1.67; 95% CI, 1.15–2.42). With respect to secondary outcomes, there were no differences between the ART and SC in the perinatal composite, any composite components in univariate and multivariate analysis (Table 2), or other neonatal outcomes (Table 3).

**TABLE 2:** Cesarean delivery and composite perinatal outcomes comparing assisted reproductive technology to spontaneously conceived pregnancies.

	ART (*n* = 157)	Spontaneous conception (*n* = 5927)	OR (95% CI)	aOR (95% CI)^c^
Primary outcome				
Cesarean delivery	47 (29.9%)	1194 (20.2%)	1.69 (1.20–2.40)	1.67 (1.15–2.42)
Secondary outcome				
Composite perinatal outcome	12 (7.6%)	540 (9.1%)	0.83 (0.46–1.50)	0.88 (0.48–1.61)
Perinatal death	0 (0.0%)	3 (0.1%)	>0.99	–
Respiratory support	5 (3.2%)	303 (5.1%)	0.61 (0.25–1.50)	0.63 (0.25–1.57)
5‐min APGAR ≤ 3	1 (0.6%)	32 (0.5%)	1.18 (0.16–8.70)	1.55 (0.20–12.20)
Hypoxic ischemic encephalopathy	1 (0.6%)	33 (0.6%)	1.15 (0.16–8.42)	1.66 (0.21–13.02)
Seizure	0 (0.0%)	15 (0.3%)	>0.99	–
Infection^a^	0 (0.0%)	21 (0.4%)	>0.99	–
Meconium aspiration	0 (0.0%)	43 (0.7%)	0.63	–
Birth trauma^b^	5 (3.2%)	244 (4.1%)	0.77 (0.31–1.89)	0.79 (0.32–1.98)
Intracranial or subgaleal hemorrhage	0 (0.0%)	16 (0.3%)	>0.99	–
Hypotension requiring vasopressor support	0 (0.0%)	7 (0.1%)	>0.99	–

*Note*: Data are presented as *n* (%), mean ± SD unless otherwise stated.

Abbreviations: aOR, adjusted odds ratio; ART, assisted reproductive technology; CI, confidence interval; OR, odds ratio.

^a^ Infection: includes pneumonia and sepsis.

^b^ Birth trauma: includes brachial plexus palsy, facial palsy, cephalohematoma, retinal hemorrhage, skull fracture, clavicular fracture, other skeletal fracture, and skin laceration.

^c^ Adjusted for advanced maternal age, treatment group, married, BMI at randomization, employment status, private insurance, and cigarette use.

**TABLE 3:** Secondary neonatal and maternal outcomes comparing assisted reproductive technology to spontaneously conceived pregnancies.

Outcome	ART (*n* = 157)	Spontaneous conception (*n* = 5927)	OR (95% CI)	aOR (95% CI)^a^
Neonatal				
Hyperbilirubinemia requiring treatment	5 (3.2%)	281 (4.7%)	0.66 (0.27–1.62)	0.68 (0.27–1.71)
Hypoglycemia	3 (1.9%)	69 (1.2%)	1.65 (0.52–5.31)	0.93 (0.22–4.00)
Admission to neonatal intermediate or intensive care unit	26 (16.6%)	723 (12.2%)	1.43 (0.93–2.19)	1.28 (0.81–2.01)
Maternal				
Type of labor				
No labor	2 (1.3%)	56 (0.9%)	1.35 (0.33–5.60)	1.34 (0.31–5.89)
Spontaneous	17 (10.8%)	907 (15.3%)	0.67 (0.41–1.12)	0.75 (0.44–1.28)
Augmented	35 (22.3%)	1509 (25.5%)	0.84 (0.57–1.23)	0.87 (0.58–1.31)
Induced	103 (65.6%)	3455 (58.3%)	1.37 (0.98–1.91)	1.30 (0.89–1.89)
Indication for cesarean delivery				
Nonreassuring fetal status	26 (41.3%)	858 (51.8%)	1.17 (0.77–1.80)	1.25 (0.80–1.95)
Labor dystocia	33 (52.4%)	709 (42.8%)	1.96 (1.32–2.90)	1.58 (1.05–2.38)
Abnormal presentation	3 (4.8%)	46 (2.8%)	2.49 (0.77–8.10)	2.13 (0.62–7.30)
Other	1 (1.6%)	42 (2.5%)	0.90 (0.12–6.57)	0.74 (0.10–5.67)
Operative vaginal delivery	16 (10.2%)	464 (7.8%)	1.34 (0.79–2.26)	1.09 (0.63–1.88)
Hypertensive disorder of pregnancy	20 (12.7%)	684 (11.5%)	1.12 (0.70–1.80)	1.57 (0.95–2.60)
Chorioamnionitis	29 (18.5%)	808 (13.6%)	1.44 (0.95–2.16)	1.41 (0.92–2.17)
Shoulder dystocia	3 (1.9%)	121 (2.0%)	0.94 (0.29–2.97)	1.25 (0.38–4.11)
Third‐ or fourth‐degree perineal laceration	7 (4.5%)	185 (3.1%)	1.45 (0.67–3.13)	1.17 (0.53–2.61)
Postpartum hemorrhage	11 (7.0%)	266 (4.5%)	1.60 (0.86–3.00)	1.67 (0.87–3.20)
Postpartum infection^a^	1 (0.6%)	114 (1.9%)	0.33 (0.05–2.36)	–
Admission to ICU	1 (0.6%)	11 (0.2%)	3.45 (0.44–26.87)	4.68 (0.47–46.26)

*Note*: Data are presented as *n* (%), mean ± SD unless otherwise stated.

Abbreviations: aOR, adjusted odds ratio; ART, assisted reproductive technology; CI, confidence interval; OR, odds ratio.

^a^ Postpartum infection: includes endometritis, pneumonia, pyelonephritis, bacteremia, and septic pelvic thrombophlebitis.

^b^ Adjusted for advanced maternal age, treatment group, married, BMI at randomization, employment status, insurance, and cigarette use.

When evaluating maternal outcomes, there were no differences in labor type; however, most patients were induced or augmented (Table 3). Additionally, there were no differences in the indication for cesarean; however, non‐reassuring fetal status and labor dystocia/arrest were the most common indications. Furthermore, there was no difference in maternal morbidities, including HDP (Table 3).

In exploratory analyses by type of ART, cesarean birth occurred in 31.1% of the IVF group, 27.8% of the OI/AI group, and 20.2% of the SC group. This corresponds to increased odds of cesarean in the IVF group (aOR, 1.75; 95% CI, 1.11–2.75) compared to SC (referent). The OI/AI group did not have significantly higher odds of cesarean (aOR, 1.52; 95% CI, 0.82–2.84). Additionally, 7.8% of patients in the IVF group, 7.4% of the OI/AI group, and 9.1% of the SC group experienced the perinatal composite outcome; however, no between‐group differences were noted (*p* = 0.010). Further, there was no difference in the perinatal composite or other neonatal morbidities comparing IVF to SC (Appendix 1). Among the groups, there were differences in cesarean indication (*p* = 0.003); however, similar to the primary analysis, no maternal morbidity differences were seen between the groups. Notably, there remained no difference in odds of HDP when comparing IVF to SC (OR, 1.44; 95% CI, 0.76–2.73; Appendix 1).

In an exploratory analysis comparing IVF pregnancies to all others, results mirrored the primary analysis. Cesarean was increased (aOR, 1.73; 95% CI, 1.10–2.72), but there was no increase in the neonatal composite outcome (aOR, 0.93; 95% CI, 0.44–1.95) or other perinatal outcomes (Appendix 2).

## 4 DISCUSSION

In this secondary analysis of ARRIVE trial, we found there was an increased incidence of cesarean in nulliparous patients undergoing term delivery who conceived with ART when compared with those who conceived spontaneously. This finding held true when comparing pregnancies conceived via specifically IVF and SC, and by IVF and all other conception methods. Additionally, findings were reassuring that there was no increased risk of HDP or other maternal or neonatal outcomes.

### 4.1 Results and clinical and research implications

Limited prior data indicate that nulliparas at term who conceived by IVF may have an increased relative risk of cesarean compared to SC, though this is higher for induced rather than expectantly managed pregnancies [6]. Our data imply that cesarean rates for ART‐conceived pregnancies are higher than SC facing term delivery. From the existing literature and currently considered analysis, an explanation for these findings remains elusive. We excluded patients with placenta previa, vasa previa, or multiple gestations and controlled for potential confounders associated with cesarean, including advanced maternal age, BMI, and smoking, which could otherwise explain the association. One potential theory is that the underlying differences in placentation with ART‐conceived pregnancies, which are implicated in HDP, are also responsible for abnormal intrapartum perfusion, resulting in abnormal fetal heart rate tracings, and thus an increased cesarean rate. Another proposed explanation is the “precious baby” effect, which references an implicit assumption on the part of the obstetrician that a patient who has tried harder or put more time or resources into conception may be more averse to even remote risks in labor, and thus leads providers to have a lower threshold for cesarean birth [7]. While this is a dangerous assumption from the ethical standpoints of patient autonomy and patient justice, it is also important for obstetricians to recognize the possibility of bias in their care of ART pregnancies. It is also possible that despite our adjusted models, there is additional unmeasured confounding due to IVF‐specific variables not included in the dataset (indication for IVF, type of transfer, day of transfer, etc.). Thus, we caution that we are not proffering a biologic or social explanation for the findings, as this is an area that needs further study.

A particularly interesting secondary finding of this study was a lack of difference in the rate of HDP between groups. It is worth noting, however, that IVF has been associated with increased rates of early‐onset and severe phenotypes of HDP [8], and subjects falling into this category would be ineligible for the ARRIVE trial.

### 4.2 Strengths and limitations

Notable strengths of our study include rigorous data collection as a secondary analysis of a multicenter trial, inclusion of sensitivity analysis by type of ART, robust multivariable analysis, and exclusion of multiple gestations and PTB. Important limitations of our study include a smaller number of patients in the ART cohort than the SC group; lack of available data regarding the indication for IVF (e.g., infertility, preimplantation genetic testing, or parental elect); and a lack of available data on IVF characteristics, including use of donor egg as well as fresh versus frozen embryo transfer, which have been shown to have a potential impact on the risk of cesarean delivery and HDP.

## 5 CONCLUSION

In our study of nulliparas delivering a singleton fetus at term, we found that ART‐conceived gestations had an increased rate of cesarean, with no difference in HDP or neonatal outcomes. Further study in a larger ART population and exploration of the mechanistic etiology and possibility of physician bias in this mode of delivery difference is warranted.

## CONFLICT OF INTEREST STATEMENT

The authors declare no conflicts of interest.

## Data Availability

Data are reproducible through the Maternal‐Fetal Medicine Units Network.

## APPENDIX 1

### 1.1

A1, A2, A3

**TABLE A1:** Baseline characteristics of pregnancies conceived via in vitro fertilization versus ovulation induction and/or artificial insemination versus spontaneous in the ARRIVE trial 2014–2017.

Characteristic	IVF (*n* = 103)	OI or AI (*n* = 54)	Spontaneous conception (*n* = 5927)	*p* value
Maternal age^a^ (years)	32.3 ± 4.1	29.3 ± 3.7	24.6 ± 4.8	<0.001
Age ≥ 35 years	35 (34.0%)	5 (9.3%)	210 (3.5%)	<0.001
Parity				<0.001
0	65 (63.1%)	38 (70.4%)	4505 (76.0%)	
1	29 (28.2%)	11 (20.4%)	1048 (17.7%)	
2	6 (5.8%)	2 (3.7%)	265 (4.5%)	
3	2 (1.9%)	2 (3.7%)	72 (1.2%)	
4	0 (0.0%)	1 (1.9%)	20 (0.3%)	
≥5	1 (1.0%)	0 (0.0%)	17 (0.3%)	
Race/Ethnicity				<0.001
Black, non‐Hispanic	4 (4.0%)	2 (3.7%)	1394 (23.7%)	
White, non‐Hispanic	82 (82.0%)	38 (70.4%)	2560 (43.5%)	
Hispanic	5 (5.0%)	8 (14.8%)	1655 (28.1%)	
Asian	9 (9.0%)	6 (11.1%)	176 (3.0%)	
Other	0 (0.0%)	0 (0.0%)	96 (1.6%)	
BMI at randomization	29.3 ± 4.9	32.0 ± 6.3	31.6 ± 6.2	<0.001
Married or living with partner	99 (96.1%)	50 (92.6%)	3453 (58.3%)	<0.001
Employment status				<0.001
Employed full time	80 (78.4%)	38 (71.7%)	2310 (39.1%)	
Employed part time	4 (3.9%)	5 (9.4%)	682 (11.5%)	
Not employed	18 (17.7%)	10 (18.9%)	2920 (49.4%)	
Private insurance	97 (94.2%)	48 (88.9%)	2587 (43.7%)	<0.001
Smoked cigarettes	0 (0.0%)	3 (5.6%)	461 (7.8%)	0.011
Drank alcohol	3 (2.9%)	3 (5.6%)	231 (3.9%)	0.62
Treatment group				0.50
Induction of labor	56 (54.4%)	30 (55.6%)	2968 (50.1%)	
Expectant management	47 (45.6%)	24 (44.4%)	2959 (49.9%)	

*Note*: Data are presented as *n* (%), mean ± SD unless otherwise stated.

Abbreviations: AI, artificial insemination; IVF, in vitro fertilization; OI, ovulation induction.

^a^Deidentified ages were omitted.

**TABLE A2:** Primary outcome and perinatal composite outcomes among pregnancies conceived via in vitro fertilization versus ovulation induction and/or artificial insemination versus spontaneous.

	IVF (*n* = 103)	OI or AI (*n* = 54)	Spontaneous conception (*n* = 5927)	
Outcome	*n* (%) aOR (95% CI)^a^	*n* (%) aOR (95% CI)^a^	*n* (%) Ref	*p* value
Cesarean delivery	32 (31.1%)	15 (27.8%)	1194 (20.2%)	0.010
	1.75 (1.11–2.5)	1.52 (0.82–2.84)	—	
Composite perinatal outcome	8 (7.8%)	4 (7.4%)	540 (9.1%)	0.82
	0.92 (0.44–1.95)	0.80 (0.29–2.23)	—	
Perinatal death	0 (0.0%)	0 (0.0%)	3 (0.1%)	>0.99
Respiratory support	4 (3.9%)	1 (1.9%)	303 (5.1%)	0.48
	0.82 (0.29–2.28)	0.33 (0.05–2.43)	—	0.48
5 min APGAR ≤ 3	0 (0.0%)	1 (1.9%)	32 (0.5%)	0.34
Hypoxic ischemic encephalopathy	1 (1.0%)	0 (0.0%)	33 (0.6%)	0.59
Seizure	0 (0.0%)	0 (0.0%)	15 (0.3%)	>0.99
Infection	0 (0.0%)	0 (0.0%)	21 (0.4%)	>0.99
Meconium aspiration	0 (0.0%)	0 (0.0%)	43 (0.7%)	>0.99
Birth trauma	3 (2.9%)	2 (3.7%)	244 (4.1%)	0.95
	0.83 (0.13–5.17)	1.13 (0.27–4.70)	—	
Intracranial or subgaleal hemorrhage	0 (0.0%)	0 (0.0%)	16 (0.3%)	>0.99
Hypotension requiring vasopressor support	0 (0.0%)	0 (0.0%)	7 (0.1%)	>0.99

*Note*: Data are presented as *n* (%), aOR (95% CI) unless otherwise stated. Spontaneous conception is the referent.

Abbreviations: AI, artificial insemination; aOR, adjusted odds ratio; CI, confidence interval; IVF, in vitro fertilization; OI, ovulation induction.

^a^Models are adjusted for advanced maternal age, treatment group, married, BMI at randomization, employment status, insurance, and cigarette use.

**TABLE A3:** Secondary neonatal and maternal outcomes among pregnancies conceived via in vitro fertilization versus ovulation induction and/or artificial insemination versus spontaneous.

	IVF (*n* = 103)	OI or AI (*n* = 54)	Spontaneous conception (*n* = 5927)	
Outcome	*n* (%) aOR (95%CI)^b^	*n* (%) aOR (95% CI)^b^	Ref	*p* value
Neonatal				
Hyperbilirubinemia requiring treatment	4 (3.9%)	1 (1.9%)	281 (4.7%)	0.75
	0.87 (0.31–2.44)	0.37 (0.05–2.72)	—	
Hypoglycemia	2 (1.9%)	1 (1.9%)	69 (1.2%)	0.32
	0.69 (0.09–5.27)	1.41 (0.19–10.47)	—	
Admission to neonatal intermediate or	18 (17.5%)	8 (14.8%)	723 (12.2%)	0.23
intensive care unit	1.30 (0.75–2.25)	1.24 (0.58–2.67)	—	
Maternal				
Type of labor				
No labor	1 (1.0%)	1 (1.9%)	56 (0.9%)	0.56
	1.00 (0.13–7.79)	1.99 (0.26–15.02)	—	
Spontaneous	10 (9.7%)	7 (13.0%)	907 (15.3%)	0.26
	0.66 (0.33–1.30)	0.93 (0.41–2.14)	—	
Augmented	26 (25.2%)	9 (16.7%)	1509 (25.5%)	0.34
	1.01 (0.62–1.64)	0.63 (0.30–1.34)	—	0.34
Induced	66 (64.1%)	37 (68.5%)	3455 (58.3%)	0.16
	1.25 (0.79–1.97)	1.41 (0.75–2.68)	—	
Indication for cesarean delivery				
Nonreassuring fetal status	24 (53.3%)	2 (11.1%)	858 (51.8%)	0.003
	1.91 (1.17–3.12)	0.25 (0.06–1.05)	—	
Labor dystocia	20 (44.4%)	13 (72.2%)	709 (42.8%)	0.002
	1.41 (0.84–2.37)	1.91 (1.00–3.64)	—	
Abnormal presentation	0 (0.0%)	3 (16.7%)	46 (2.8%)	0.015
Other	1 (2.2%)	0 (0.0%)	42 (2.5%)	0.68
Type of Analgesia/Anesthesia				
None	1 (1.0%)	0 (0.0%)	119 (2.0%)	0.71
IV/IM	21 (20.4%)	18 (33.3%)	2179 (36.8%)	0.003
	0.57 (0.35–0.94)	0.94 (0.52–1.69)	—	
Epidural/ Combined spinal‐epidural	80 (77.7%)	36 (66.7%)	3557 (60.0%)	<0.001
	1.74 (1.08–2.81)	1.17 (0.65–2.10)	—	
Spinal	1 (1.0%)	0 (0.0%)	60 (1.0%)	>0.99
General	0 (0.0%)	0 (0.0%)	11 (0.2%)	>0.99
Operative vaginal delivery	13 (12.6%)	3 (5.6%)	464 (7.8%)	0.16
	1.35 (0.73–2.49)	0.61 (0.19–1.97)	—	
Hypertensive disorder of pregnancy	12 (11.7%)	8 (14.8%)	684 (11.5%)	0.76
	1.44 (0.76–2.73)	1.81 (0.83–3.92)	—	
Chorioamnionitis	19 (18.5%)	10 (18.5%)	808 (13.6%)	0.22
	1.36 (0.81–2.31)	1.51 (0.75–3.04)	—	
Shoulder dystocia	1 (1.0%)	2 (3.7%)	121 (2.0%)	0.44
	0.71 (0.10–5.28)	2.01 (0.47–8.55)	—	
Third‐ or fourth‐degree perineal laceration	5 (4.9%)	2 (3.7%)	185 (3.1%)	0.39
	1.24 (0.48–3.20)	1.04 (0.25–4.32)	—	0.39
Postpartum hemorrhage	9 (8.7%)	2 (3.7%)	266 (4.5%)	0.12
	2.14 (1.03–4.45)	0.86 (0.21–3.58)	—	0.12
Postpartum infection^a^	1 (1.0%)	0 (0.0%)	114 (1.9%)	0.79
Admission to ICU	1 (1.0%)	0 (0.0%)	11 (0.2%)	0.27

*Note*: Data are presented as *n* (%), mean ± SD, and aOR (95% CI) unless otherwise stated. Spontaneous conception is the referent.

Abbreviations: AI, artificial insemination; aOR, adjusted odds ratio; CI, confidence interval; IVF, in vitro fertilization; OI, ovulation induction.

^a^ Postpartum infection: includes endometritis, pneumonia, pyelonephritis, bacteremia, and septic pelvic thrombophlebitis.

^b^ Models are adjusted for advanced maternal age, treatment group, married, BMI at randomization, employment status, insurance, and cigarette use.

## APPENDIX 2

### 2.1

B1, B2, B3

**TABLE B1:** Baseline characteristics of pregnancies conceived via in vitro fertilization versus non–in vitro fertilization among low‐risk nulliparous women delivering in the ARRIVE trial 2014–2017.

Characteristic	IVF (*n* = 103)	Non‐IVF (*n* = 5981)	*p* value
Maternal age^a^ (years)	32.3 ± 4.1	24.6 ± 4.9	<0.001
Age ≥ 35 years	35 (34.0%)	215 (3.5%)	<0.001
Total number of previous pregnancies			0.030
0	65 (63.1%)	4543 (76.0%)	
1	29 (28.2%)	1059 (17.7%)	
2	6 (5.8%)	267 (4.5%)	
3	2 (1.9%)	74 (1.2%)	
4	0 (0.0%)	21 (0.4%)	
≥5	1 (1.0%)	17 (0.3%)	
Race/Ethnicity			<0.001
Black, non‐Hispanic	4 (4.0%)	1396 (23.5%)	
White, non‐Hispanic	82 (82.0%)	2598 (43.8%)	
Hispanic	5 (5.0%)	1663 (28.0%)	
Asian	9 (9.0%)	182 (3.1%)	
Other	0 (0.0%)	96 (1.6%)	
BMI at randomization	29.3 ± 4.9	31.6 ± 6.2	<0.001
Married or living with partner	99 (96.1%)	3503 (58.6%)	<0.001
Employment status			<0.001
Employed full time	80 (78.4%)	2348 (39.4%)	
Employed part time	4 (3.9%)	687 (11.5%)	
Not employed	18 (17.7%)	2930 (49.1%)	
Insurance status			<0.001
Private	97 (94.2%)	2635 (44.1%)	
Public/Self‐pay	6 (5.8%)	3345 (55.9%)	
Smoked cigarettes	0 (0.0%)	464 (7.8%)	0.003
Drank alcohol	3 (2.9%)	234 (3.9%)	0.80
Treatment group			0.39
Induction of labor	56 (54.4%)	2998 (50.1%)	
Expectant management	47 (45.6%)	2983 (49.9%)	

*Note*: Data are presented as *n* (%), mean ± SD unless otherwise stated.

Abbreviation: IVF, in vitro fertilization.

^a^ Deidentified ages were omitted.

**TABLE B2:** Primary outcome and perinatal composite outcomes among pregnancies conceived via in vitro fertilization versus non–in vitro fertilization.

Outcome	IVF (*n* = 103)	Non‐IVF (*n* = 5981)	OR (95% CI)	aOR (95% CI)^a^
Cesarean delivery	32 (31.1%)	1209 (20.2%)	1.78 (1.17–2.71)	1.73 (1.10–2.72)
Composite perinatal outcome	8 (7.8%)	544 (9.1%)	0.84 (0.41–1.74)	0.93 (0.44–1.95)
Perinatal death	0 (0.0%)	3 (0.1%)	>0.99	–
Respiratory support	4 (3.9%)	304 (5.1%)	0.76 (0.28–2.06)	0.83 (0.30–2.31)
5 min APGAR ≤ 3	0 (0.0%)	33 (0.6%)	>0.99	–
Hypoxic ischemic encephalopathy	1 (1.0%)	33 (0.6%)	1.77 (0.24–13.05)	2.88 (0.3–23.41)
Seizure	0 (0.0%)	15 (0.3%)	>0.99	–
Infection	0 (0.0%)	21 (0.4%)	>0.99	–
Meconium aspiration	0 (0.0%)	43 (0.7%)	>0.99	–
Birth trauma	3 (2.9%)	246 (4.1%)	0.70 (0.22–2.22)	0.74 (0.23–2.39)
Intracranial or subgaleal hemorrhage	0 (0.0%)	16 (0.3%)	>0.99	–
Hypotension requiring vasopressor support	0 (0.0%)	7 (0.1%)	>0.99	–

*Note*: Data are presented as *n* (%), mean ± SD unless otherwise stated.

Abbreviations: aOR, adjusted odds ratio; CI, confidence interval; IVF, in vitro fertilization; OR, odds ratio.

^a^ Models are adjusted for advanced maternal age, treatment group, married, BMI at randomization, employment status, insurance, and cigarette use.

**TABLE B3:** Secondary neonatal and maternal outcomes among pregnancies conceived via in vitro fertilization versus non–in vitro fertilization.

Outcome	IVF (*n* = 103)	Non‐IVF (*n* = 5981)	OR (95% CI)	aOR (95% CI)^b^
Neonatal				
Hyperbilirubinemia requiring treatment	4 (3.9%)	282 (4.7%)	0.82 (0.30‐2.23)	0.88 (0.31‐2.48)
Hypoglycemia	2 (1.9%)	70 (1.2%)	1.67 (0.41–6.91)	0.69 (0.09–5. 22)
Admission to neonatal intermediate or intensive care unit	18 (17.5%)	731 (12.2%)	1.52 (0.91–2.54)	1.29 (0.75–2.24)
Maternal				
Type of labor				
No labor	1 (1.0%)	57 (1.0%)	1.02 (0.14–7.43)	0.98 (0.13–7.62)
Spontaneous	10 (9.7%)	914 (15.3%)	0.60 (0.31–1.15)	0.66 (0.33–1.31)
Augmented	26 (25.2%)	1518 (25.4%)	0.99 (0.63–1.55)	1.02 (0.63–1.65)
Induced	66 (64.1%)	3492 (58.4%)	1.27 (0.85–1.91)	1.24 (0.79–1.95)
Indication for cesarean delivery				
Nonreassuring fetal status	24 (53.3%)	860 (51.4%)	1.81 (1.14–2.87)	1.94 (1.19–3.17)
Labor dystocia	20 (44.4%)	722 (43.2%)	1.76 (1.07–2.88)	1.38 (0.82–2.33)
Abnormal presentation	0 (0.0%)	49 (2.9%)	>0.99	–
Other	1 (2.2%)	42 (2.5%)	1.39 (0.19–10.17)	1.18 (0.15–9.38)
Type of Analgesia/Anesthesia				
None	1 (1.0%)	119 (2.0%)	0.48 (0.07–3.49)	0.82 (0.11–6.18)
IV/IM	21 (20.4%)	2197 (36.7%)	0.44 (0.27–0.71)	0.57 (0.35–0.94)
Epidural/ Combined spinal‐epidural	80 (77.7%)	3593 (60.1%)	2.31 (1.45–3.69)	1.74 (1.08–2.80)
Spinal	1 (1.0%)	60 (1.0%)	0.97 (0.13–7.05)	0.85 (0.11–6.59)
General	0 (0.0%)	11 (0.2%)	>0.99	–
Operative vaginal delivery	13 (12.6%)	467 (7.8%)	1.71 (0.95–3.07)	1.36 (0.74–2.51)
Hypertensive disorder of pregnancy	12 (11.7%)	692 (11.6%)	1.01 (0.55–1.85)	1.42 (0.75–2.69)
Chorioamnionitis	19 (18.5%)	818 (13.7%)	1.43 (0.86–2.36)	1.35 (0.80–2.28)
Shoulder dystocia	1 (1.0%)	123 (2.1%)	0.47 (0.07–3.37)	0.70 (0.09–5.18)
Third‐ or fourth‐degree perineal laceration	5 (4.9%)	187 (3.1%)	1.58 (0.64–3.93)	1.24 (0.48–3.19)
Postpartum hemorrhage	9 (8.7%)	268 (4.5%)	2.04 (1.02–4.09)	2.15 (1.03–4.46)
Postpartum infection^a^	1 (1.0%)	114 (1.9%)	0.51 (0.07–3.65)	0.69 (0.09–5.19)
Admission to ICU	1 (1.0%)	11 (0.2%)	5.32 (0.68–41.60)	7.22 (0.68–77.15)

*Note*: Data are presented as *n* (%), mean ± SD unless otherwise stated.

Abbreviations: aOR, adjusted odds ratio; CI, confidence interval; IVF, in vitro fertilization; OR, odds ratio.

^a^ Postpartum infection: includes endometritis, wound infection, pneumonia, pyelonephritis, bacteremia, and septic pelvic thrombophlebitis

^b^ Models are adjusted for advanced maternal age, treatment group, married, BMI at randomization, employment status, insurance, and cigarette use.
